# 

**Published:** 1923-11

**Authors:** 


### RECENT APPOINTMENTS.

### Medical Officers, Chaplains, &c.

Anderson, Gilbert, M.B., Ch.B., D.P.H., Assistant Medical School Inspector, Staffordshire.

Belam, F. A., Assistant M.O.H., Chester; M.O.H., Brighouse.

Boobbyer, Philip, M.R.C.S., M.O.H., Nottingham; Small-Pox Commissioner for the East Midlands.

Byrne, Austen W., M.B., Ch.B., D.P.H.; Assistant M.O.H. and Assistant School M.O., Bolton (Liverpool University).

Drummond, Peter, M.B., Ch.B.; Medical Superintendent Mid-Wales Mental Hospital, Talgarth.

Goddard, C. E., O.B.E., M.R.C.S., L.R.C.P., M.O.H., Harrow and Wembley; M.O., British Empire Exhibition.

Gregory, James, M.B., Ch.B., D.P.H., Assistant Medical School Inspector, Staffordshire.

Jolly, Doris E. P., M.R.C.S., L.R.C.P., D.P.H., Resident M.O., Wolverhampton and Staffordshire Hospital; Junior M.O.H., Ipswich, £500.

Macdonald, J. J., Assistant M.O.H., Wandsworth.

Mackie, Thomas Jones, M.D., D.P.H., Professor of Bacteriology, Cape Town University; Professor of Bacteriology, Edinburgh University.

McLachlan, Jessie, M.B., Ch.B., D.P.H., Assistant Medical School Inspector, Staffordshire.

Patrick, Adam, M.D., Professor of Medicine, Glasgow University.

Richardson, Barbara, M.B., Ch.B., D.P.H., Assistant Medical School Inspector, Staffordshire.

Roper, Charles, M.D., Medical Superintendent Herts County Tuberculosis Sanatorium; Tuberculosis Officer Kent (Westminster Hospital).

Turner, Adam A., M.B., Ch.B., D.P.H., M.C.; Deputy M.O.H. and School M.O., Croydon.

Turner, Mary I., M.B., Ch.B., D.P.H.; Assistant M.O.H. Chester. £500-£550.

### Public Health and Hospital Appointments.

Anthony, Eliza, Night Superintendent and Assistant Matron, Leeds City Hospitals; Matron, Infectious Diseases Hospital and Sanatorium, Darwen.

Catherwood, Maud E.,; Health Visitor, Brentford.

Cherry, P. T., Assistant Sanitary Inspector, Walton; Sanitary Inspector, Warsop.

Knox, Clyde; Assistant Sanitary Inspector, Thornaby-on-Tees; Sanitary Inspector, Thornaby-on-Tees, £225-£250.

Currie, John Ronald, M.A., M.D., D.P.H., Professor of Preventive Medicine, Queen's University, Kingston, Ontario; Professor of Public Health, University of Glasgow.

Dunkley, Ethel; Health Visitor and School Nurse, Twickenham.

McGuire, Florence, Tuberculosis Health Visitor, Hull.

Miller, Isabel, Assistant Matron, Ministry of Pensions Hospital, Richmond Park; Matron, Bolingbroke Hospital, Wandsworth Common.

Murray, J. Dick, C.M.B.; Matron, King Edward VII. Hospital, Hertford Hill.

Smith, F. W., Assistant Sanitary Inspector, Farnborough; Assistant Sanitary Inspector, Norwich, and Inspector under Corporation Market Act and Shop Acts, £130, with bonus.

